# Modelling farm-to-farm disease transmission through personnel movements: from visits to contacts, and back

**DOI:** 10.1038/s41598-017-02567-6

**Published:** 2017-05-24

**Authors:** Gianluigi Rossi, Rebecca L. Smith, Stefano Pongolini, Luca Bolzoni

**Affiliations:** 1 Department of Pathobiology, College of Veterinary Medicine, University of Illinois, 2001 S. Lincoln Avenue, 61802 Urbana, IL USA; 2 Risk Analysis Unit, Istituto Zooprofilattico Sperimentale della Lombardia e dell’Emilia-Romagna, Via dei Mercati, 13/A, I-43126 Parma, Italy

## Abstract

Infectious diseases in livestock can be transmitted through fomites: objects able to convey infectious agents. Between-farm spread of infections through fomites is mostly due to indirect contacts generated by on-farm visits of personnel that can carry pathogens on their clothes, equipment, or vehicles. However, data on farm visitors are often difficult to obtain because of the heterogeneity of their nature and privacy issues. Thus, models simulating disease spread between farms usually rely on strong assumptions about the contribution of indirect contacts on infection spread. By using data on veterinarian on-farm visits in a dairy farm system, we built a simple simulation model to assess the role of indirect contacts on epidemic dynamics compared to cattle movements (i.e. direct contacts). We showed that including in the simulation model only specific subsets of the information available on indirect contacts could lead to outputs widely different from those obtained with the full-information model. Then, we provided a simple preferential attachment algorithm based on the probability to observe consecutive on-farm visits from the same operator that allows overcoming the information gaps. Our results suggest the importance of detailed data and a deeper understanding of visit dynamics for the prevention and control of livestock diseases.

## Introduction

Highly contagious infectious diseases, such as foot-and-mouth disease (FMD), classical swine fever, and highly pathogenic avian influenza, represent major threats for the livestock industries worldwide^1, 2^. Past epidemics have caused losses of millions of dollars, and several million animals have been culled as a consequence of disease control programs^3, 4^. In order to reduce potential losses, veterinary public health agencies have focused on building surveillance systems able to quickly identify ongoing outbreaks, and set up control plans able to effectively and rapidly extinguish livestock epidemics. To this aim, scenario analyses based on knowledge of the infection spread patterns are essential^5^. In this context, mathematical and simulation-based models represent a very useful tool to generate a deeper understanding of the mechanisms underlying the disease spread processes, as well as to support decision makers in planning biosecurity measures, surveillance, and control activities.

In modelling livestock diseases, much attention has been paid to alive animal movements (i.e. direct contacts), which are considered the main route of between-farm transmission for a large number of infections^6^. Thanks to the availability of extensive databases developed in different countries to track movements of farmed animals, several studies have examined the main features of direct contacts network structure and their implications for disease spread^2, 7–11^.

However, routes of between-farm transmission other than animal movements, such as infection through fomites (i.e. contaminated clothes, equipment, and vehicles), have been found to be an important component of the spread of many diseases, including FMD^12^ in ruminants and swine, classical swine fever in swine^13^, and influenza A in both swine^14^ and poultry^15^. Fomite transmission generally occurs through farm-to-farm movements of personnel and operators (i.e. indirect contacts)^16^. The most at risk operator categories include veterinarians, artificial insemination technicians, milk trucks, transporters of livestock, and rendering trucks (which come in close contact with animals and their waste)^17, 18^. A paradigmatic case was the 2001 FMD epidemic in the UK. Despite an early animal movement ban, the infection continued to spread between farms through indirect contacts and the epidemic was eradicated months later, only after the imposition of a stringent stamping-out policy^12, 19^.

A general lack of knowledge about the potential effect of indirect contacts is mostly due to the limited availability of contact data, usually obtained through voluntary surveys of farmers and operators^16–18, 20, 21^. Alternatively, the fomites spread has been directly estimated using data from the trace-back of between-farm infections in previous outbreaks (see ref. 22, and references therein). A further approach to identify indirect contacts was to use commercial links among farms due to common contractors^23–25^. In this way, the authors overcame the temporal and spatial limitations intrinsically associated with voluntary surveys by building indirect contacts networks involving several farms. However, relying on commercial relationships could hide the sequentiality of contacts, as well as overestimate the number of links between farms, as already observed by Garcia-Alvarez *et al*.^26^. As a consequence, epidemiological simulation-based models often accounted for the effect of indirect contacts in between-farm transmission by using limited information on visitors and vehicle movements.

The first objective of this study was to quantitatively evaluate the effect of fomites on transmission of highly infectious diseases among dairy farms. The population of interest comprised all dairy farms that were producing milk for human consumption in the Province of Parma, Italy in 2013. For each dairy farm for the period from January 1^st^ to December 31^st^ we obtained detailed records of farm-to-farm movements of livestock and farm visits made by veterinarians^27^. The main features of the contact networks generated by livestock movements and veterinarian visits were investigated in a previous work^27^. Here, to evaluate the role of fomites on transmission, we built and analysed a stochastic farm-to-farm Susceptible-Infectious-Susceptible (SIS) spread model including both transmission through animal movements (i.e. direct contacts) and veterinarian on-farm visits (i.e. indirect contacts).

The second objective was to identify the essential features of the indirect contacts structure that allowed us to accurately describe between-farm spread of infection. More specifically, we ran different model simulations with each scenario including only specific subsets of the total information available to us about on-farm veterinarian visits. These information subsets were chosen to reproduce some of the common information limitations that modellers usually face. The aim of this approach was to test the assumptions that modellers make to compensate for those information deficiencies against the full-information model. Finally, we proposed a simple preferential attachment algorithm, in which the veterinarians that already visited the farm had a higher probability of being re-assigned to the following visits in the same farm.

The ultimate goal of this work was to provide a proof-of-principle that different assumptions made to describe indirect contacts in models for livestock diseases can lead to significantly different outcomes from the epidemiological point of view. As such, the study was not intended to accurately describe the spread of a specific disease in livestock, but to identify general features on indirect contacts that can be applied in different epidemiological contexts.

## Methods

### Data and study system

The study area was the province of Parma, in Emilia Romagna region (Northern Italy). This area accounted for *N* = 1,349 dairy farms active during year 2013. Dairy farm data included the farm’s unique identification code and geographical locations^27^. Cattle movement data were obtained from the Bovine National Database (BDN, Commission Decision 2006/132/EC) and each of the 16,647 individual between-farm records included the unique animal identifier, origin farm code, destination farm code, and movement date^27^.

Indirect contacts were estimated from the on-farm visits of two personnel categories: veterinary officers and practitioners. Veterinary officers consisted of veterinarians in service at the Parma Province Local Health Unit (LHU), while veterinary practitioners consisted of veterinarians hired or requested directly by farm managers to take care of their animals. The officer and practitioner datasets included respectively 6,524 and 14,053 unique on-farm visits, for a total of 20,577 visits combined. Further data details, as well as a network analysis of both direct and indirect contacts network were described by Rossi *et al*.^27^.

### Epidemic model

The disease spread within the Parma Province’s dairy system was represented by a simple Susceptible-Infectious-Susceptible (SIS) compartmental model. We assumed farms as discrete single units, as other theoretical studies of the spread of highly contagious infectious diseases through animal movements^2^. Other than the initial infected farm, all farms were assumed to be susceptible at the beginning of each simulation. At each time step (assumed one day), an infectious farm could transmit the disease through direct or indirect contacts. A direct contact occurred when at least one animal was moved from an infected farm to a susceptible one, and the probability of direct contact infection was *β*_*dir*_. Indirect contact transmission is due to the spread of infected fomites, carried farm-to-farm by operators (specifically veterinarians). In our model, we assumed that veterinarians might become contaminated by visiting an infected farm. Then, they were able to spread the infection to susceptible farms visited afterwards, with probability of infection *β*_*ind*_. We defined a contamination period *h* as the average pathogen survival time in fomites, and thus the time period in which a veterinarian was able to potentially infect susceptible farms. Infectious farms returned to a susceptible state after an infectious period of *γ* days. The infectious period represents the time-period farms stay infected, and it should not be confused with the individual infectious period (representing the time period individuals stay infected).

### Epidemic simulations

The described model was used to run a set of simulations of epidemics within the study system. The contribution of the indirect contacts was assessed by testing seven different values of *β*_*ind*_ across the range [0, 1]. We assumed a deterministic effect of the direct contacts transmission (i.e. *β*_*dir*_ = 1)^2^. The infectious period *γ* was set at 14 days. The ability of pathogens to survive in fomites is strictly pathogen-dependent and can be variable depending on the contaminated surface and on environmental conditions^28^. In the case of FMD, fomites are believed to be capable of lasting up to several days, although precise estimates are difficult in real environmental settings, while experimental trials were often performed in laboratories. We set *h* at 14 days (2 weeks), as previously assumed in the case of FMD^29–31^. To generalize our findings to diseases with different epidemiological features with respect to FMD, we performed sensitivity analyses for parameters *γ* and *h*, which are included in the Supplementary material S1.

For each scenario, we simulated 5,000 runs. In each run we randomly selected the initial seed (i.e., the farm in which the epidemic started) and the potential epidemic starting day. The latter was done to include temporal variability in the simulated epidemics: the initial day was randomly selected among the first 4 months of year 2013 (January 1^st^–April 30^th^), while simulations were extended for either a maximum period of 245 days (≈ 8 months) or until the epidemic faded out, whichever happened first. For each run, we randomly selected the configuration of the indirect contacts network with regard to the simulated within-day farm visits order, to address the uncertainty due to the missing within-day visit sequence as in Rossi *et al*.^27^. Their results showed that the network structure was not substantially affected by the variability in the within-day veterinarian itineraries.

### Number of potentially infectious contacts generated by each visit

We analysed the distribution of the number of contacts generated by each individual visit, for a given value of *h*. We fitted different theoretical distributions (e.g. power-law, discrete exponential, Poisson log-normal, and Poisson) using the Maximum Likelihood Estimation technique. For each value of *h*, we selected the best models and the minimum threshold value (*x*_*min*_) using the Vuong’s Test^32, 33^. For each of the tested distributions we also performed a sensitivity analysis on *x*_*min*_. We evaluated a range of potential lower bound values between 1 and the observed 80^th^ quantile, to include at least 20% of the observations (see Supplementary material Fig. S2.1). We used the Kolmogorov-Smirnov (KS) test to evaluate the goodness-of-fit^33^.

Further, we investigated the effects on the epidemiological dynamics generated by the assumption of considering the number of on-farm visits as a suitable proxy of the indirect contacts generated by veterinarians. Specifically, we ran a set of simulations using an epidemic model in which each visit corresponded to exactly one potentially infectious contact, referred to as one-contact-per-visit model. The destination farm was selected depending on the distance from the contact source (see next paragraph for details).

### Simulation models with rewired contact networks

To understand the features of the indirect contact networks that are essential to appropriately describe between-farm disease spread, we investigated the infection dynamics through simulated networks built with a subset of the total information available on the veterinarians’ on-farm visits. We ran different sets of simulations for as many alternative contact networks in which we held the total number of indirect contacts generated by each visit as observed in the data, but, for each contact generated by visits in a source farm, we randomly rewired the between-farm contacts choosing the destination farms following different selection rules^34^. Specifically, we built random networks with the same number of farms as in the observed indirect contact network, but we randomly allocated the edges between the nodes. In this way, the farms degree distribution (i.e. the network linking pattern on disease spread) was held as in the observed network.

In the first set of simulations, the destination farm of each contact generated by visits in a source farm was selected among the farms belonging to set ***Q***_***k***_(***h***) with equal probability, where ***Q***_***k***_(***h***) represents the set of farms that received a visit between the day *k* and the following *h* days, referred to as the random rewire (RR) model.

In the second set of simulations, the destination farm of each contact generated by visits in a source farm was randomly selected through a distance dependent rewiring, referred to as the distance-dependent rewire (DR) model. Again, the destination farm of each contact generated by a visit occurred at day *k* was randomly selected within set ***Q***_***k***_(***h***). Differently from the RR model, in RD model the probability *PS*_*ijk*_(*h*) of farm *j* of being selected as a destination of a contact generated from a visit in farm *i* occurred at day *k* was equal to:

1 $$P{S}_{ijk}(h)=\{\begin{array}{c}\frac{P({d}_{ij})}{{\sum }_{j}P({d}_{ij})}\,{\rm{i}}{\rm{f}}\,j\in {Q}_{k}(h)\\ 0\,\,{\rm{o}}{\rm{t}}{\rm{h}}{\rm{e}}{\rm{r}}{\rm{w}}{\rm{i}}{\rm{s}}{\rm{e}}\end{array},$$

where *P*(*d*_*ij*_) corresponds to the probability to observe a contact between farms *i* and *j* located at distance *d*_*ij*_, computed as:

2 $$P({d}_{ij})=\frac{exp(\alpha +\delta {d}_{ij})}{1+exp(\alpha +\delta {d}_{ij})}.$$

with parameters α and δ estimated by fitting a logistic regression model on the observed pattern of indirect contacts as function of the inter-farm distance, *d*_*ij*_.

In the third set of simulations, the destination farm of each contact generated by visits in a source farm was randomly selected among the farms that belonged to the same veterinarian-defined cluster as the source farm, referred to as the clustered rewired (CR) model. As farms were often visited by more than one veterinarian, we used the Jaccard Index to calculate a similarity value for each pair of farms as follows:

3 $$J{I}_{ij}=\frac{|{V}_{j}\cap {V}_{i}|}{|{V}_{j}\cup {V}_{i}|},$$

where ***V***_*i*_ represents the set of veterinarians visiting farms *i*, and ***V*** *j* the set of veterinarians visiting farm *j* during the observation period. Here, the probability of a farm *j* to be selected as a destination of a contact starting from farm *i* was:

4 $$P{C}_{ijk}(h)=\{\begin{array}{c}\frac{J{I}_{ij}}{{\sum }_{j}J{I}_{ij}}\,{\rm{i}}{\rm{f}}\,j\in {Q}_{k}(h)\\ 0\,\,{\rm{o}}{\rm{t}}{\rm{h}}{\rm{e}}{\rm{r}}{\rm{w}}{\rm{i}}{\rm{s}}{\rm{e}}\end{array}.$$

Then, in the RC model, a higher number of veterinarian in common between two farms corresponded to a higher probability to assign a potentially infectious contact between the farms.

### Simulation models with preferential attachment algorithm

Finally, we tested whether tracking the identity of farm visitors (through the veterinarian identifiers) in a partial-information model can improve the epidemiological predictions. Specifically, we run two sets of epidemic simulations in which each on-farm visit was held as in the original dataset, but the identifier of the veterinarians performing the visit was randomly assigned. The assignments were done in two alternative ways: firstly, by drawing the veterinarian identifiers with probability proportional to the frequency of the visits observed in the original dataset; secondly, by using a preferential attachment criteria to assign a veterinarian identifier to each visit. In the first set of simulations, each visit in a given farm was independently assigned. In the second set of simulations, the first visit in a given farm is assigned as in the previous case, while all the non-first-time visits had a larger probability to be assigned to the veterinarians having already visited the same farm. This probability was computed in the original dataset as the proportion of the non-first-time visits performed by a veterinarian having already visited the same farm, over the sum of non-first-time visits in each farm.

## Results

### On the transmission probability of indirect contacts

**Figure 1:**
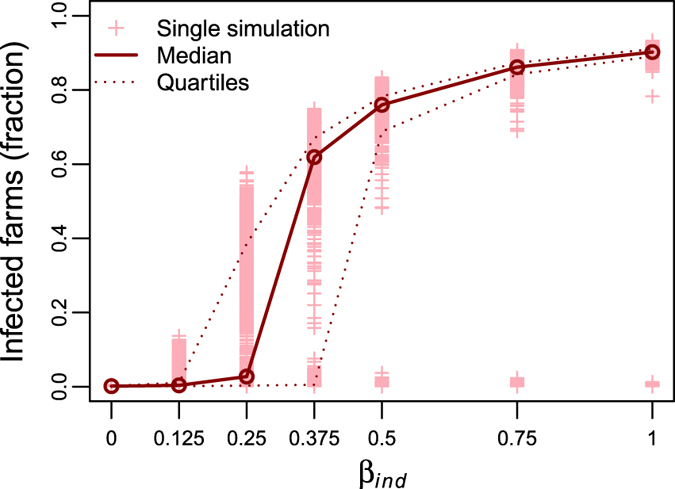
*β*_*ind*_ sensitivity analysis. The distribution of the total epidemic size from 5,000 simulations for six values of *β*_*ind*_: 0, 0.125, 0.25, 0.375, 0.5, 0.75, and 1. Crosses represent the fraction of infected farms in each simulation, the thick line the median of the infected farms fraction for the epidemics with secondary cases, and the dotted lines the 25^th^ and 75^th^ quantiles.

To understand the role of indirect contacts on epidemic dynamics, we ran simulations of the stochastic epidemic model assuming that direct contacts always generate an infection in the receiving farm (i.e. probability of infection *β*_*dir*_ = 1) and testing a range of probability values for between-farm transmission due to indirect contacts (*β*_*ind*_). Specifically, the simulation outputs considered were: (*i*) the fraction of runs in which secondary cases were generated (i.e., number of total infected farms was larger than 1), as shown in Table 1; and (*ii*) the proportion of infected farms in the overall network in epidemic simulations where secondary cases occurred, as shown in Fig. 1. When *β*_*ind*_ = 0 (i.e. only direct contacts generate infections), only in 8.0% of the simulated epidemics secondary cases occurred. However, the number of simulations with secondary infected farms rapidly increased with *β*_*ind*_ and reached a plateau of about 40% for values of *β*_*ind*_ larger than 0.25 (see Table 1). Among epidemics having secondary cases, Fig. 1 shows that the proportion of infected farms significantly increased with *β*_*ind*_: from a few infected farms per epidemic when *β*_*ind*_ = 0, to the majority of the simulated epidemics involving over 60% of the farms when *β*_*ind*_ was larger than 0.25. Specifically, Fig. 1 highlights the presence of a sort of percolation threshold at *β*_*ind*_ ≈ 0.25, above which epidemics can spread in a large portion of the farm system. Additionally, for values of *β*_*ind*_ lower than 0.25, almost all simulated epidemics faded out within the simulation period (8 months), generating no or few secondary cases at worst.

**Table 1:** *β*_*ind*_ sensitivity analysis indicators.

β_IND_	β_DIR_	Outbreaks with secondary cases					Self-extinct epidemics
		Fraction	Size				
			25^th^ percentile	Median	75^th^ percentile	Max	
0.0	1	8.0%	2	2	3	8	99.9%
0.125	1	32.7%	2	5	15	185	98.5%
0.25	1	38.3%	4	37	520	780	81.5%
0.375	1	40.8%	7	835	904	1011	72.0%
0.5	1	42.6%	933	1025	1056	1129	66.5%
0.75	1	44.0%	1136	1162	1178	1225	61.8%
1	1	44.0%	1201	1217	1228	1259	60.8%

Indicators for the indirect contact transmission probability *β*_*ind*_ sensitivity analysis based on 5,000 runs for each parameter defined scenario. We showed the percentage of epidemics with secondary cases, and for those epidemics the 25^th^, 50^th^ (median), 75^th^ quantiles and the maximum, and finally the number of self-extinct epidemics.

Sensitivity analyses of the epidemiological parameters used in the simulations, namely the contamination period (*h*) and the farms’ infectious period (*γ*), are reported in the Supplementary material S1. Our analyses on *h* and *γ* showed that values of the epidemiological parameter might quantitatively change the model outputs, especially when the value of *β*_*ind*_ is low, suggesting that appropriate estimates of pathogen survival in fomites and farm environment are important for understanding livestock infection dynamics.

### On the number of contacts generated by on-farm visits

**Figure 2:**
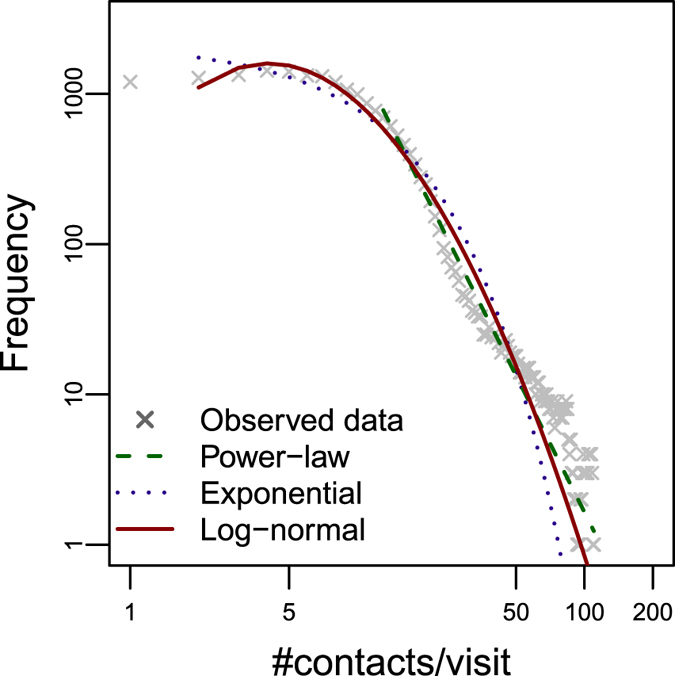
Distribution of the number of contacts generated by visits. Distribution (on a log-log scale) of the number of contacts per visit (*h* = 14 days). Crosses represent the observed data, thick line: discrete log-normal fit, dotted line: discrete exponential fit, dashed line: discrete power law fit (distributions fitted using the maximum likelihood estimation^32, 52^).

In the previous section, we showed that indirect contacts could substantially contributes to farm disease spread. The epidemiological explanation of our finding relies on the peculiar contact structure generated by consecutive on-farm visits. In this section, we showed that a single on-farm visit could generate a large number of potentially infectious contacts among farms within the time-period where fomites stay contaminated (specifically, 14 days), as shown in Fig. 2. The Kolmogorov-Smirnov (KS) test to evaluate the goodness-of-fit^33^ showed that Poisson log-normal distribution provided a better fit of the data. However, for high values of *x*_*min*_ (≥13), the Vuong’s test applied to the comparison between Poisson log-normal and power law distributions provided p-values > 0.05, meaning that the tail of the distribution can be appropriately described by both Poisson log-normal and power law. The analysis of the distribution of contacts per visit under different assumptions on the values of the contamination period, *h*, is reported in the Supplementary material, section S2. From the analyses follows that the distribution of potentially infectious contacts per visit can be well described by heavy-tailed distributions (such as, Poisson-lognormal and power law), suggesting that there exists a small number of on-farm visits potentially responsible for a large number of contacts, thus playing the role of super-spreader events in the epidemic dynamics.

### On the use of on-farm visit frequencies to estimate potentially infectious contacts

**Figure 3:**
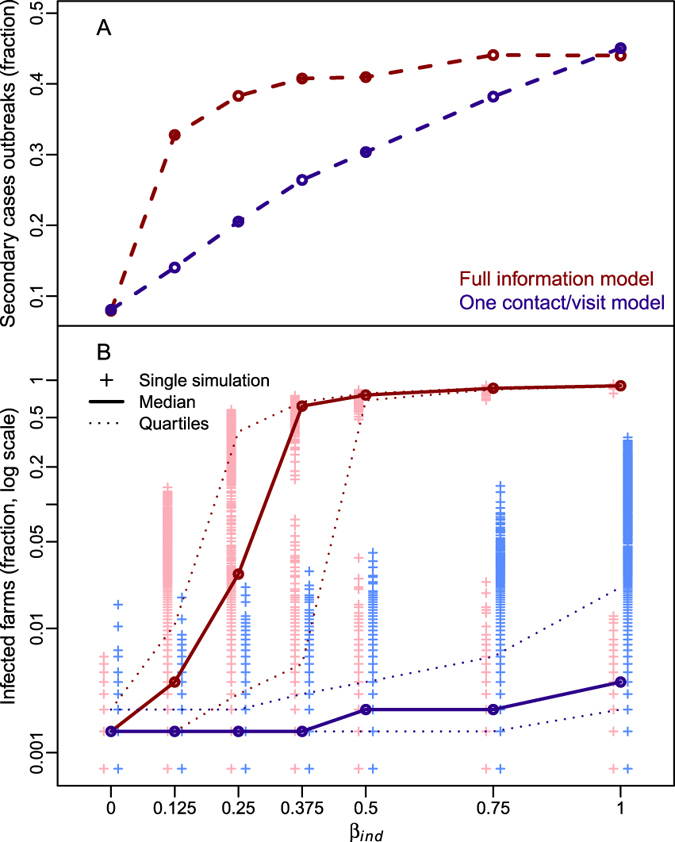
Epidemic simulations assuming one-contact-per-visit model. Results of the simulations comparing the full-information model (magenta), the model assuming only one contact generated by each visit (blue). Panel A: dashed lines represent the fraction of epidemics with secondary cases. Panel B: the distribution of the number of infected farms (crosses), with the median (thick line), and the 25^th^ and 75^th^ quantiles (dotted lines). *h* is set to 14 days.

Estimates of the frequency distribution of personnel on-farm visits have been previously used to parameterize visitor-mediated contacts in simulation-based models for livestock diseases. This approach is equivalent to assume that each on-farm visit generates a single potentially infectious contact, here referred to as one-contact-per-visit model. Here, we compared the simulation outputs of epidemics generated through the observed contact network against simulations generated under the assumption that each on-farm visit generates a single potentially infectious contact. As showed in Fig. 3, the outcomes were substantially different between the full-information model (magenta lines) and the one-contact-per visit model (blue lines). When the contamination period (*h*) was set to 14 days, the one-contact-per-visit model substantially underestimated the epidemic sizes across all values of *β*_*ind*_. For instance, at *β*_*ind*_ = 0.25, median [inter-quartile range] of infected farms was 0.15% [0.15–0.22%] vs. 2.74% [0.29–38.55%] in the full-information model.

### On the simulations with rewired contact networks

**Figure 4:**
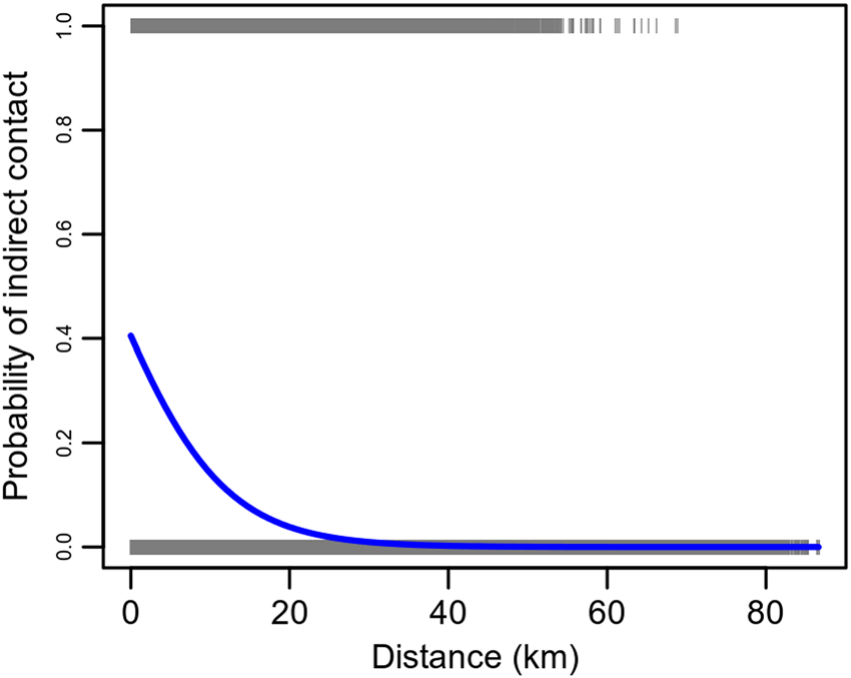
Probability of indirect contacts vs. distance. Logistic regression model for indirect contacts (*h* = 14 days) vs. distance. The grey bar represents the observed contacts, the blue line the fitted model.

Here, we tested the assumptions that the knowledge of: (*i*) the distribution of contacts per visit (as in Fig. 3), RR model; (*ii*) inter-farm distances, DR model; and (*iii*) veterinarian-defined clusters, CR model, provide sufficient information to reproduce epidemic features observed with the model built with full information. For the DR model, we estimated the role of inter-farm distance on the probability to observe indirect contacts through a logistic regression model. The logistic regression on the farm-to-farm contact data provided by the veterinarian visits showed a significant negative effect of inter-farm distance on the indirect contact occurrences (p-values < 0.001). In particular, Fig. 4 showed that the probability to observe contacts is very low for distances larger than 20 km.

For the CR model, we computed the Jaccard Index matrix as in (3). As shown in Fig. S3.1, the Jaccard Index matrix was generally sparse, with the exception of four recognizable farm subgroups. These subgroups correspond to the four geographical areas of responsibility (called districts) of the Local Health Unit (LHU) of Parma Province, since each veterinary officer usually operates in a single district.

**Figure 5:**
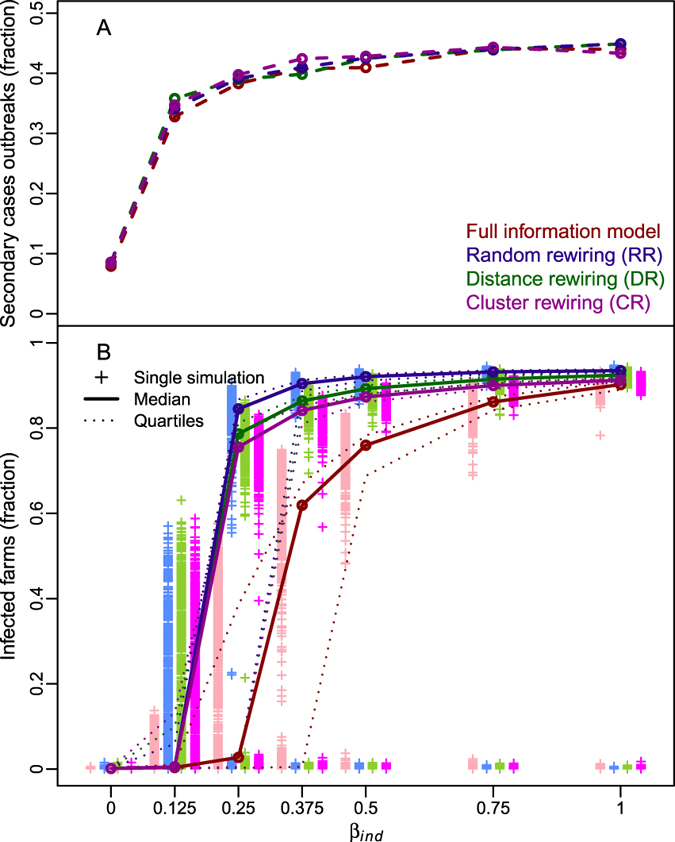
Epidemic simulations with indirect contacts rewiring models. Results of the simulations comparing the full-information model (magenta) with the distance rewired (DR, green), random rewired (RR, blue), and clustered rewired (CR, purple) indirect contacts models. Panel (A) dashed lines represent the fraction of epidemics with secondary cases. Panel (B) the distribution of the number of infected farms (crosses), with the median (thick line), and the 25^th^ and 75^th^ quantiles (dotted lines).

The comparison of the epidemic dynamics obtained with the full-information model (magenta lines and crosses) with the RR (blue lines and crosses), DR (green lines and crosses), and CR (purple lines and crosses) models is shown in Fig. 5. While the fraction of simulated epidemics generating secondary cases was the same between simulations using rewired and full-information models (see panel A in Fig. 5), simulations using RR, DR and CR models significantly overestimated epidemic sizes predicted by the full information model (magenta lines and crosses in Fig. 5) when secondary cases occurred (see panel B in Fig. 5).

### On the simulations with a preferential attachment algorithm

**Figure 6:**
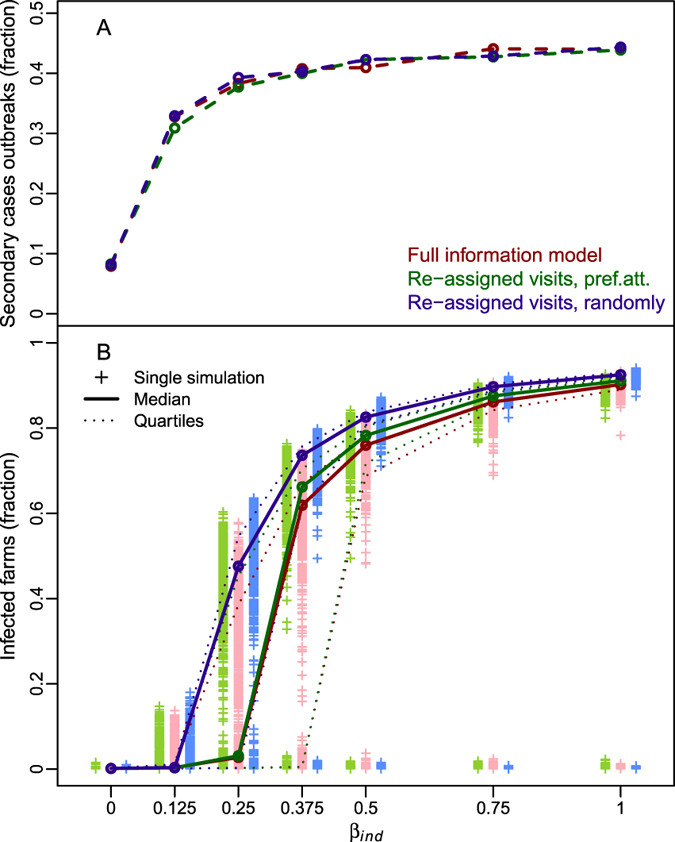
Epidemic simulations with preferential attachment visits assignment. Results of the simulations comparing the full-information model (magenta), and two alternative set of simulations. We assigned the on-farm visits to veterinarians randomly (blue) and following a preferential attachment criteria (green). Panel (A) dashed lines represent the fraction of epidemics with secondary cases. Panel (B) the distribution of the number of infected farms (crosses), with the median (thick line), and the 25^th^ and 75^th^ quantiles (dotted lines).

In the previous section, we showed that all partial-information models failed in accurately describing the epidemic dynamics, suggesting that the lack of information affecting these models actually limited their ability to reproduce the behaviour of the real contact network. To investigate what component could bridge the gap between the partially-informed networks and the real one, we ran two further sets of simulations randomly assigning a veterinarian identifier to each visit. As we showed in Fig. 6, by assigning the visits to randomly chosen veterinarians (blue lines and crosses) the fraction of simulated epidemics producing secondary cases was more similar to the full-information model outcomes (magenta line, see panel A). However, as for all previous analysed models, the epidemic size was generally overestimated (see Panel B). Alternatively, on-farm visits were assigned using a preferential attachment criteria. Using this approach, the first visit to a farm was randomly assigned, then the probability of selecting a veterinarian that had already visited the farm was set to 77% (calculated for the observed visits, see Methods section). In this case, both the fraction of outbreaks with secondary cases (see panel A in Fig. 6, green line) and the epidemic size (see Panel B in Fig. 6, green lines and crosses) were very similar to the full-information model outcomes.

## Discussion

The main scope of our work was to understand the role of different between-farm transmission routes in infectious disease spread, with a particular focus on contacts due to personnel movements. Our results showed that transmission of infection arising from on-farm visits by veterinarians might substantially enhance the epidemic spread and sustain major outbreaks in a population of dairy farms. On the one hand, this result might have been intuitive as, by including indirect contacts, we increased the total number of potentially infectious contacts within the system. On the other hand, our simulations showed that major epidemics were observed when the probability of infection associated to indirect contacts (*β*_*ind*_) exceeded a given threshold (i.e. *β*_*ind*_ > 0.25, Fig. 1), a substantially lower value than the probability of infection associated with direct ones (*β*_*dir*_). This result was consistent with the findings previously obtained comparing the structure of the direct and indirect contacts networks in the same area of study^27^.

Our analyses showed that, in the absence of high-resolution data on on-farm visits, the assumptions made on the indirect contact structure to describe transmission in simulation models can lead to substantially different epidemic dynamics.

Firstly, we highlighted the need for clear and distinct definitions for on-farm visits and farm-to-farm contacts, stressing that the two concepts are not overlapping. Assuming that one visit corresponds to a unique potentially infectious contact can produce misleading results. On the one hand, when the contamination period (*h*) was long, e.g. 14 days, the assumption that each visit produces a potentially infectious contact resulted in a significant underestimation of the epidemic sizes. On the other hand, for very short *h*, e.g. when only the within-day visits can result in a potentially infectious contact, the epidemic sizes were overestimated (see Fig. S2.4 in the Supplementary material). These effects on the estimation of epidemic size were a consequence of the high heterogeneity observed in the number of contacts generated by each visit. Despite the distribution of the number of contacts per visit was substantially dependent on the assumptions made on the contamination period length (*h*), the distribution showed similar characteristics across the evaluated range of values (Supplementary material S2). In particular, in all the investigated cases, the distribution was suitably fitted by Poisson log-normal and power law distributions. This result suggested that the number of contacts per visit can be described through heavy-tailed distributions, with crucial consequences on the between-farm infection dynamics (as shown in several works on heterogeneous networks^35–37^). On the one hand, heavy-tailed distributions of contacts might increase the stochastic effects of a potential epidemic spread, leading to a higher number of self-extinctions. On the other hand, they might also increase the epidemic potential by significantly reducing the epidemic threshold for disease invasion and persistence^38^.

Secondly, we showed that simply knowing the distribution of potentially infectious contacts generated by each visit does not provide sufficient information to accurately describe between-farm infectious dynamics. Simulations performed through rewired contact networks with different destination-selecting rules failed to replicate the dynamics observed in the full-information model. Despite the median distance of the observed indirect contacts being significantly lower than the median between-farm distance (see Fig. 4), RR and DR model outcomes were not substantially different. In the past, different simulation models for livestock diseases included disease spread mechanisms in which the probability for a susceptible farm to become infected was inversely proportional to the distance to the infected farm^18, 22^. On the one hand, these models were useful because they could include a variety of infection mechanisms that are difficult to be tracked (such as personnel’ visits, local contacts, and airborne spread). On the other hand, we observed that at small spatial scales such as the Parma Province, the inter-farm distance might not be a good covariate to describe the indirect contacts between farms generated by personnel movements.

In the clustered rewired model (CR), the destination farms were chosen only among those visited by the same veterinarians in the original dataset (Fig. S3.1). The CR model was based on the same logic of epidemiological network models built using commercial links between farms to infer contacts^23–25^. These networks were built linking farms that share suppliers from commercial enterprises operating in the livestock sector (such as feed manufacturers, egg or milk collectors, slaughterhouses, multi-site companies, live animal markets). Similarly, in the CR model the farms could be linked only when they shared at least one veterinarian, without taking into account the time-interval between on-farm visits and the daily itineraries. This approach was very useful to identify general trends among large networks of farms. However, our results showed that such as approach, by neglecting the spatio-temporal sequence of veterinarian visits, overestimated the size of a potential epidemic in a dairy system.

Simulations ran with models built by randomly assigning the veterinarian identifiers to the observed on-farm visits, showed similar outcomes to those produced using the full-information model. In particular, we showed that accounting for the recurring visits by the same veterinarian (i.e. assuming preferential attachment mechanisms) was crucial to accurately reproduce disease dynamics. Similarly to the findings obtained by Valdano *et al*.^39^ for cattle movements, our results suggested that a certain level of loyalty between veterinarians and farms appears to have a significant effect in epidemic spread.

Previous studies showed that an effective strategy for disease control in heterogeneous networks would be targeting the most at-risk nodes (defined as super-spreaders)^34^. Studies on cattle movement networks have confirmed the effectiveness of removing high contact farms^37^, or manipulating the network to disrupt the most at-risk links^40^. However, given the general lack of information on indirect contacts, the definition of the most at-risk farms and links might be difficult. Usually, the best prevention and control measures in relation to indirect contacts consist in maintaining an overall high level of hygiene and biosecurity, as these might disrupt dangerous contacts^12^. Practices such as avoiding equipment sharing or promoting equipment disinfection, positioning of vehicle bath systems, providing individual protection equipment and boot cleaners, and limiting visitors have already proven to be very effective in reducing between-farm disease transmission^41^. Here, we presented a modelling approach where the risk associated to each farm to spread infections via indirect contacts can be evaluated in a simulation framework, providing a useful tool to effectively direct investigations on the biosecurity level towards the more at-risk farms.

Our results suggested that, in livestock diseases, indirect contacts due to personnel visits can be better represented by modelling methods describing infectious links using personnel itineraries, rather than the distribution of between-farm contacts and/or inter-farm distances. In the case of simulation-based models for FMD spread, either the personnel itineraries^42^ or the distribution of contacts^30, 43–46^ have been implemented. The comparison of the predictions obtained by different FMD models showed that their outcomes differed more in term of disease spread than in term of the effectiveness of disease control strategies^47, 48^. This suggests that improvements in the description of indirect contacts can be beneficial when models are developed as tools for epidemiological descriptions more than when they are developed as support for decision-making.

Our work is based on a too simplistic framework to evaluate the effectiveness of disease-specific models, such as FMD. On the other hand, the simplicity of the framework allowed us to draw more general conclusions about between-farm contacts that can be tested in more sophisticated and disease-specific models. In particular, our framework lacks different epidemiological aspects which are crucial for livestock diseases spread. First, following Bajardi *et al*.^2^, our model accounted only for the between-farm dynamic of the epidemic. Previous works showed that even for fast spreading diseases, the within farm dynamic is very important in order to make good predictions^19^. In fact, only accounting for the delay between the infection entering a farm and the onset of the infectivity, as well as for different levels of infectivity through time, can substantially change the outcome of simulation models^19^. Second, we analysed the spread only within the dairy farms, while the analysis of other important farming systems, such as beef farms and swine farms are essential for bovine (the former) and multi-species (the latter) diseases spread. Third, we used movement data for animals and personnel obtained when no epidemic was ongoing in the study area, implying that the observed patterns can provide a good prediction of the infectious contacts before the detection of an epidemic, while we can expect different patterns once the infection has been detected.

The databases used in this study represent only a small fraction of the on-farm visits due to external personnel and operators. In fact, the analyses did not take into account regular commercial activities - such as milk and feed trucks, hoof trimmers, dead haulers, artificial insemination technicians - that would significantly increase the potentially infectious contacts and, consequently, the number of secondary infections and the epidemic sizes^49^. Despite each type of contact features specific patterns (e.g. regularity and length of the itineraries), the general nature of our results on personnel itineraries makes the study findings potentially extendible to the various visitor and personnel activities that might be involved in disease transmission.

Sensitivity analyses on the pathogen survival in fomites (*h*), or contamination period, and the farm infectious period (*γ*) showed their major influence on simulation outcomes. This is of substantial importance considering the variability of these two parameters. The contamination period, *h*, mostly depends on the pathogen’s ability to survive in fomites, but it is also strongly affected by environmental factors and biosecurity measures^18^. Experiments on *Mycobacterium bovis* ^28^ and on FMD virus^29^ showed a high variability in the pathogen’s survival period, depending on the type of substrate and to the environmental conditions, such as temperature and humidity. Predictably, our analysis showed an effect of *h* on the infectious dynamics, in particular on the epidemics size (Fig. S1.1). Notably, by assuming a 21 days contamination period, the percolation threshold, originally observed at *β*_*ind*_ ≈ 0. 25 when *h* = 14 days, was set to *β*_*ind*_ ≈ 0.125, while when *h* was reduced to 7 days, epidemics were able to expand to a major portion of the network only for *β*_*ind*_ ≥ 0.75.

The value of *γ* is disease-specific and it depends on the within-farm disease dynamics, in particular on host-to-host transmission and host infectious period. The value used in this work, 14 days, can represent the farm infectious period associated to acute contagious diseases, such as FMD^30^ (although, when control measures such as quarantine or depopulation are applied, the period in which a farm stays infectious can be even shorter^50^). Our sensitivity analysis on *γ* values showed a clear shift of the epidemiological outcomes for higher values of farm infectious period (S1.2). As this parameter strictly depends on the within-farm epidemic dynamics, this result suggested potentially different behaviours of diseases characterized by different dynamics.

Animal movements are with no doubt the most effective route of disease transmission between-farm^6^, however, our work highlighted a general need for a deeper knowledge of transmission mediated by fomites, including better ways to properly capture the frequency and probability of contacts due to personnel movements on- and off- farm^49^. In this respect, improved data collection and analyses would be crucial to better understand the impact of this phenomenon in cattle as well as in poultry and swine industries. The increasing demand for meat and dairy products from human populations, and the consequent growing global livestock industry, makes livestock epidemics a major threat for animal and human health, as well as for the livestock industry^51^. Thus, a better understanding of the roles and effects of each epidemic transmission route is more urgent than ever. Our work represents a further step in the direction of a deeper knowledge of the between-farm transmission phenomenon.

## Electronic supplementary material

Supplementary material
