# Supplementary material for: Modelling farm-to-farm disease transmission through personnel movements: from visits to contacts, and back
Source: Sci Rep. 2017 May 24;7:2375. doi: 10.1038/s41598-017-02567-6 (PMC5443770; doi:10.1038/s41598-017-02567-6)
Supplement: Supplementary file 1 — Supplementary material [file 41598_2017_2567_MOESM1_ESM.pdf]

Supplementary material

Modelling farm-to-farm disease transmission through personnel movements: from visits to contacts, and back

G. Rossi, R. L. Smith, S. Pongolini, and L. Bolzoni

S1. Sensitivity analyses

In this section, we provided sensitivity analyses for the epidemiological parameters used in the stochastic model: the veterinarians contamination period and the farms infectious period, indicated as h and γ, respectively.

In the original model both parameters were set to 14 days (see Methods section in main text). For the sensitivity analysis of the contamination period, we performed simulations assuming a low (7 days) and a high (21 days) value. Since the value of farms infectious period used in the main text was already on the low boundary for the more common livestock diseases, we performed sensitivity analysis using higher values of γ: 28 and 42 days. Results are shown in Figures S1.1 and S1.2, respectively.

S2. Number of contacts per visit

In the main text we showed the results of the analysis of the distribution of the indirect contacts generated by veterinarians' visits (Figure 2). In Figure S2.1, we showed the best fitting sensitivity analysis for different values of x_{min}, which represents the distribution's heavy-tailed behaviour positive lower bound (i.e. > 0).

Here, we performed the analysis of the distribution of the number of contacts per visit provided in the main text assuming four alternative contamination period lengths: 0, 7, 21, and 28 days. We showed the results of the fitting procedures in Figure S2.2, while in Figure S2.3 we showed the results of the Kolmogorov-Smirnov and Vuong's tests for a range of x_{min} values.

In Table S2.1 we reported the values of the number of contacts per visit distribution for all the considered values of h. Moreover, we showed the parameters of the best fitted discrete log-normal and power-law distributions, as these are the distributions which provided the best fit to the data.

In the main text we showed how a simulation model that assumed one contact per visits was unable to produce similar epidemic dynamics compared with a the simulation model using the original data (main text Figure 3). To evaluate the potential effect of the contamination period on this result, we repeated the analysis for an h value of 0 days (as opposed to the benchmark of 14 days); this was equivalent to assuming that potentially infectious contacts only occur within the same day of the original infected farm visit. As showed in Figure S2.4, when $h = 0$, the simulation model assuming one indirect contact per visit overestimated the extent of a potential epidemic, in contrast to the $h = 14$ case (see main text Figure 3) in which the outbreaks extent were underestimated.

S3. JI matrix and visits per veterinarian alternative distributions

In Figure S3.1 we showed the Jaccard Index matrix, in a grey scale gradient (white correspond to 0, black correspond to 1). For each pair of farms *i* and *j* their JI_{ij} was calculated following the Jaccard index as showed in the Methods section of the main text. The values of the *JI* matrix were used as probability for two farm to be have an indirect contact in the cluster rewiring model (CR).

In the main text, we presented an analysis in which we investigated the role of the veterinarian-farm relationship. We re-assigned the observed on-farm visits to different veterinarians in two ways, randomly and following a preferential attachment criteria (i.e. it is more likely for a farm to be visited by a veterinarian that already visited it, see main text for details). In these, we maintained the distribution of the number of visits per veterinarian by assuming that each veterinarian did the same exact number of visits that we observed in our dataset. As showed in Figure S3.2, this distribution was very skewed. To test the potential role of this, we did a further analysis in which we assumed two alternative distributions for the number of visits per veterinarian: uniform and normal. One example for each distribution was showed in Figure S3.3. Using the previous criteria, we firstly assigned to each veterinarian a new number of visits, and then we coupled the veterinarians with the observed on-farm visits following the preferential attachment algorithm. As in the analysis presented in the main text, the first visit in each farm was assigned to a random veterinarian, while from the second on there was a 77% probability to be assigned to a veterinarian that had already visited the farm (see main text's Method section for details on this probability calculation). Thus, we simulated the epidemic spread within the system using the indirect contacts obtained as described.

As showed in Figure S3.4, by using alternative visits per veterinarian distributions we failed to reproduce the same patterns observed in the original simulations. In both cases, the extent of an epidemic was underestimated, as well as the number of outbreaks able to generate secondary cases.

Figures and tables

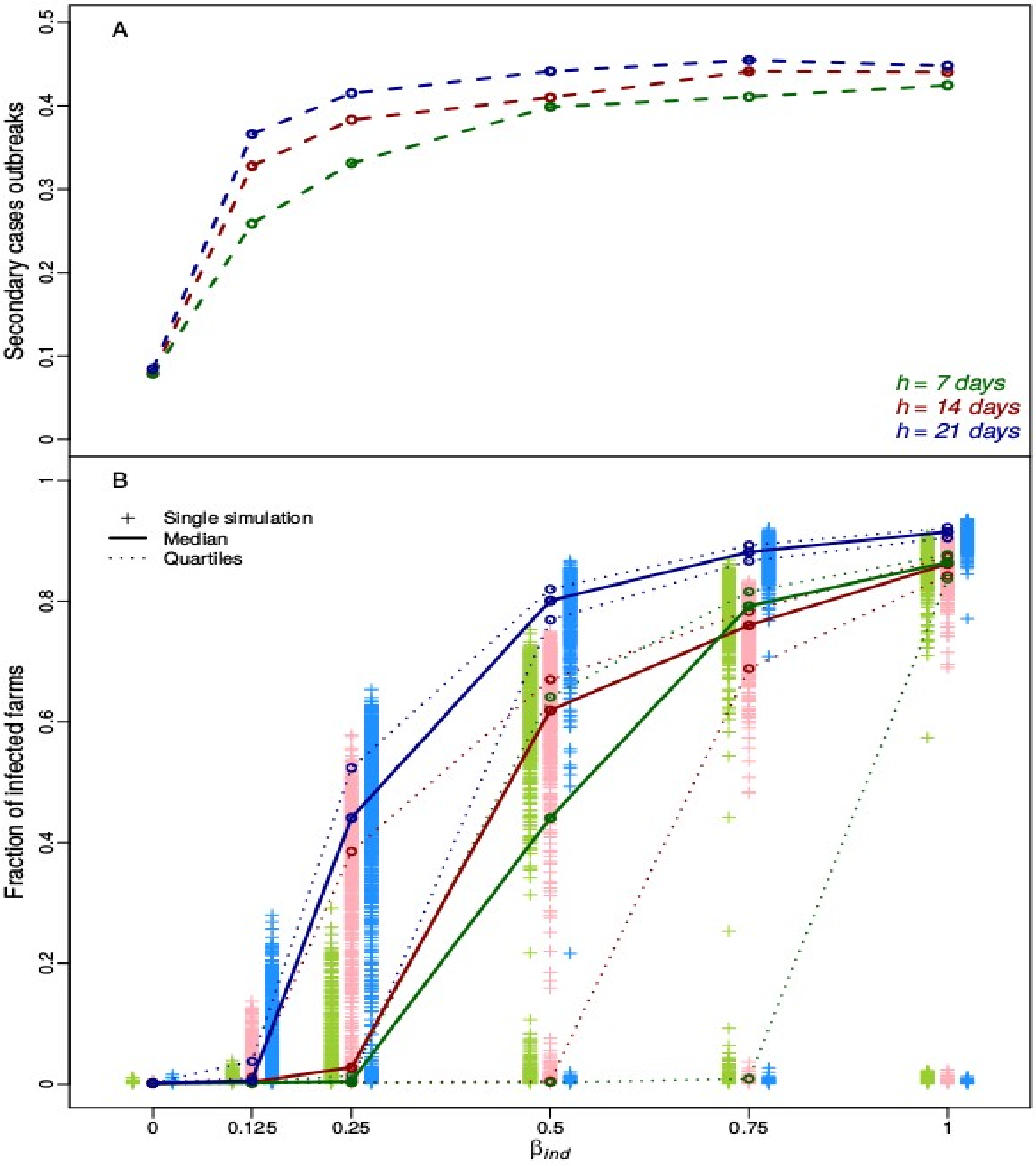

Figure S1.1: Contamination period (*h*) sensitivity analysis.

The effect of *h* value on simulations outcome: 7 (green), 14 (red, benchmark), and 21 (blue) days. Panel A: fraction of outbreaks with secondary cases. Panel B: crosses represent the fraction of infected farms in each simulation, the thick line represents the median, and the dotted lines represent the inter-quartile range.

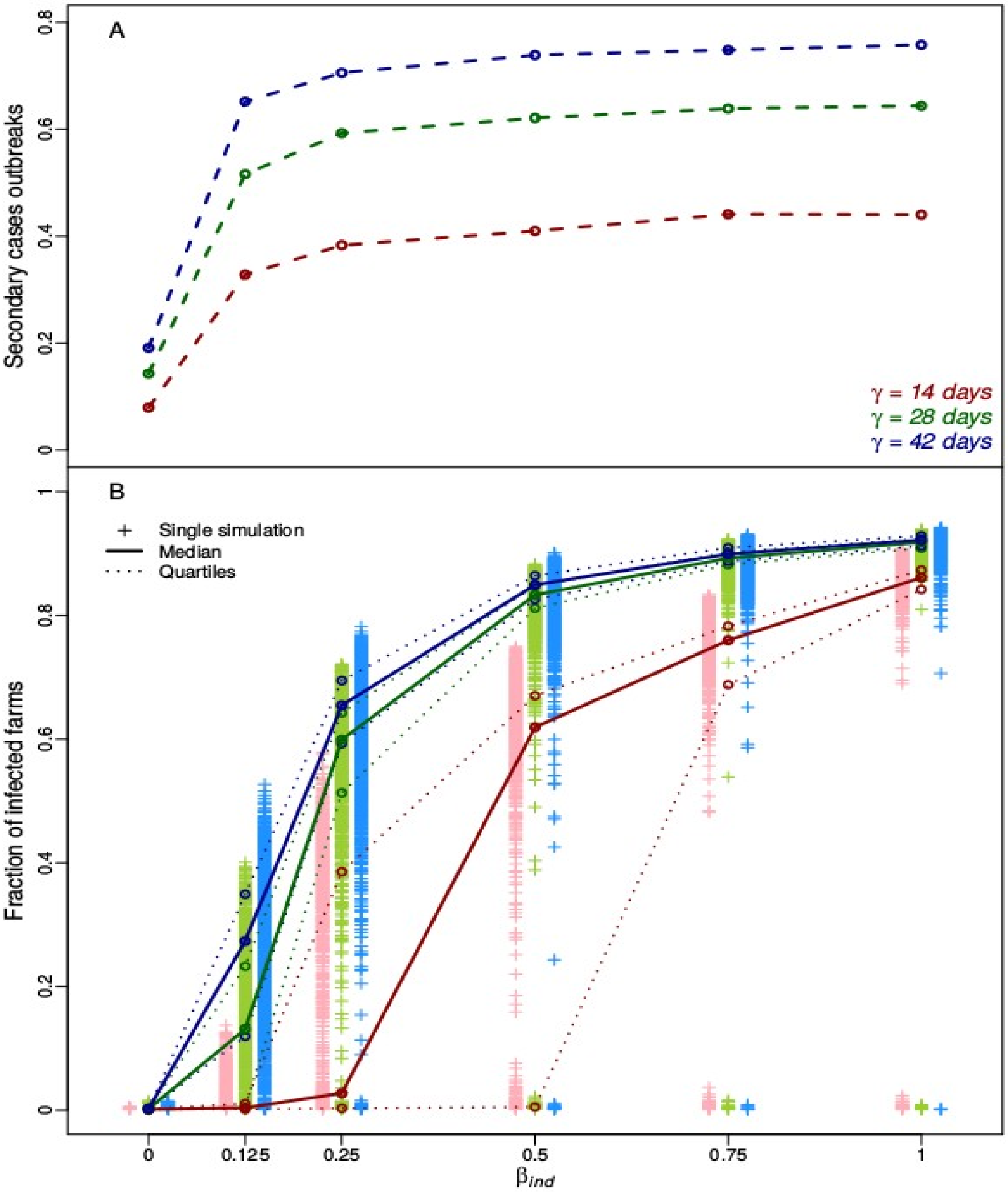

Figure S1.2: Infectious period (γ) sensitivity analysis.

The effect of γ value on simulations outcome: 14 (red, benchmark), 28 (green), and 42 (blue) days. Panel A: fraction of outbreaks with secondary cases. Panel B: crosses represent the fraction of infected farms in each simulation, the thick line represents the median, and the dotted lines represent the inter-quartile range.

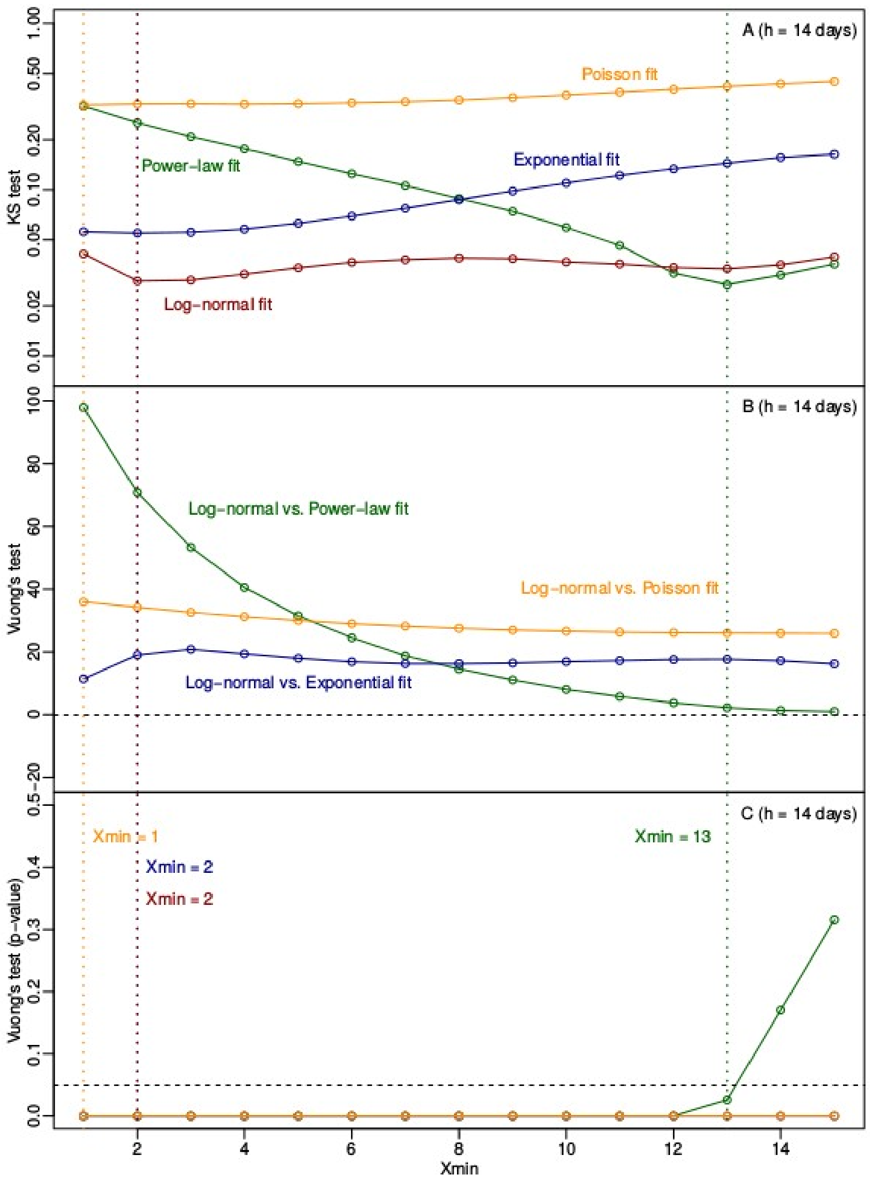

Figure S2.1: Sensitivity analysis of x_{min} .

Comparison of four theoretical distribution functions fitting (Poisson log-normal, discrete exponential, power law, and Poisson) on the contact per visits data under the assumption of h = 14 days. The figure shows the effect of the x_{min} threshold choice on the performance of the four considered distributions. Panel A: Kolmogorov-Smirnov goodness-of-fit test results; panel B: Vuong's test results; panel C: Vuong's test p-value.

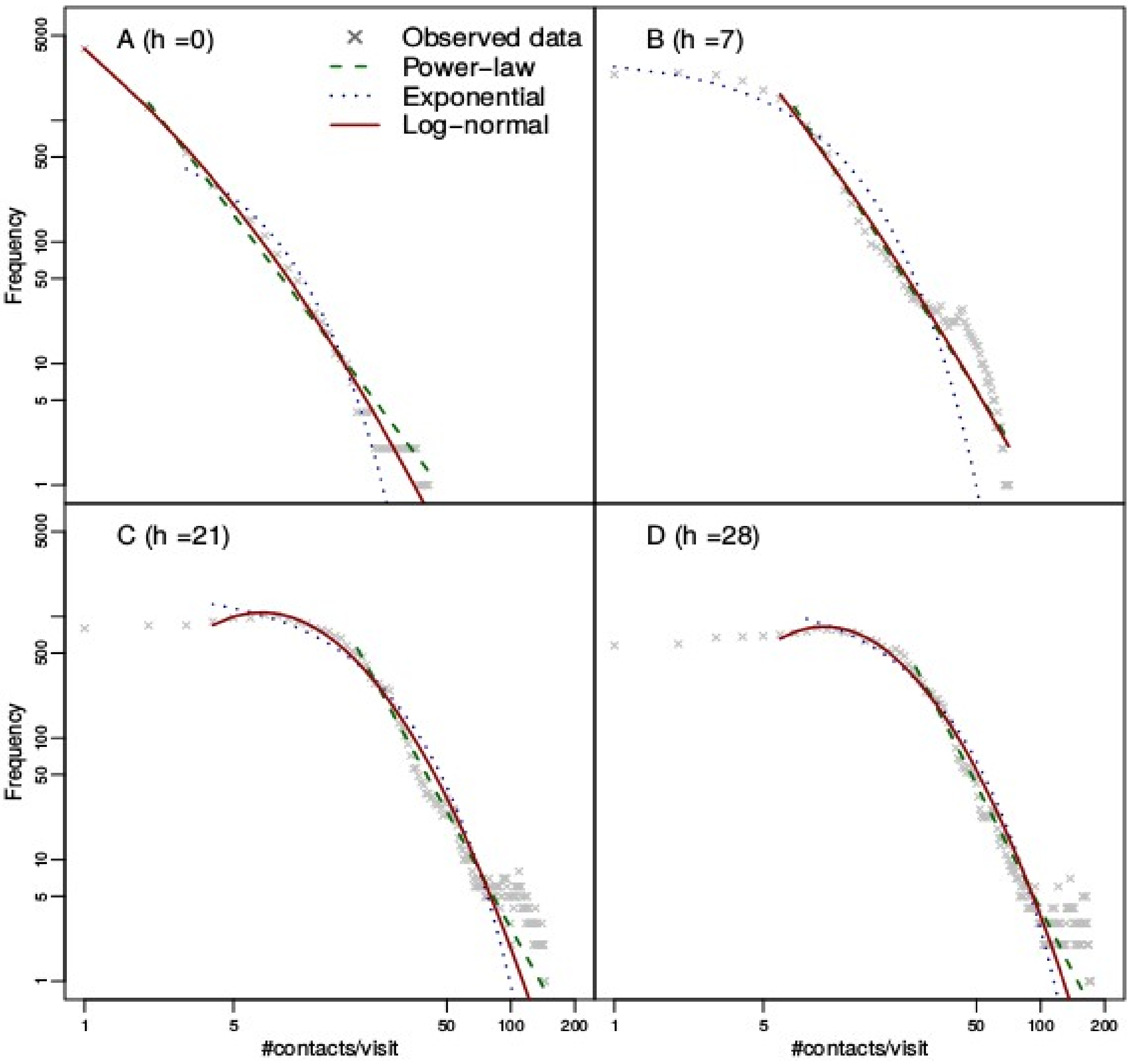

Figure S2.2: Distribution of the number of contacts generated by visits.

Crosses represent the data, thick line: Poisson log-normal fit, dotted line: discrete exponential fit, dashed-dotted line: Poisson fit, dashed line: discrete power law fit (log-log scale, only positive values, distributions fitted using the maximum likelihood estimation – Clauset et al., 2009; Gillespie, 2015). In each panel we show the fitting of the four selected distributions on the contact per visit generated with *h* set to 0 (panel A), 7 (panel B), 21 (panel C), and 28 (panel D) days.

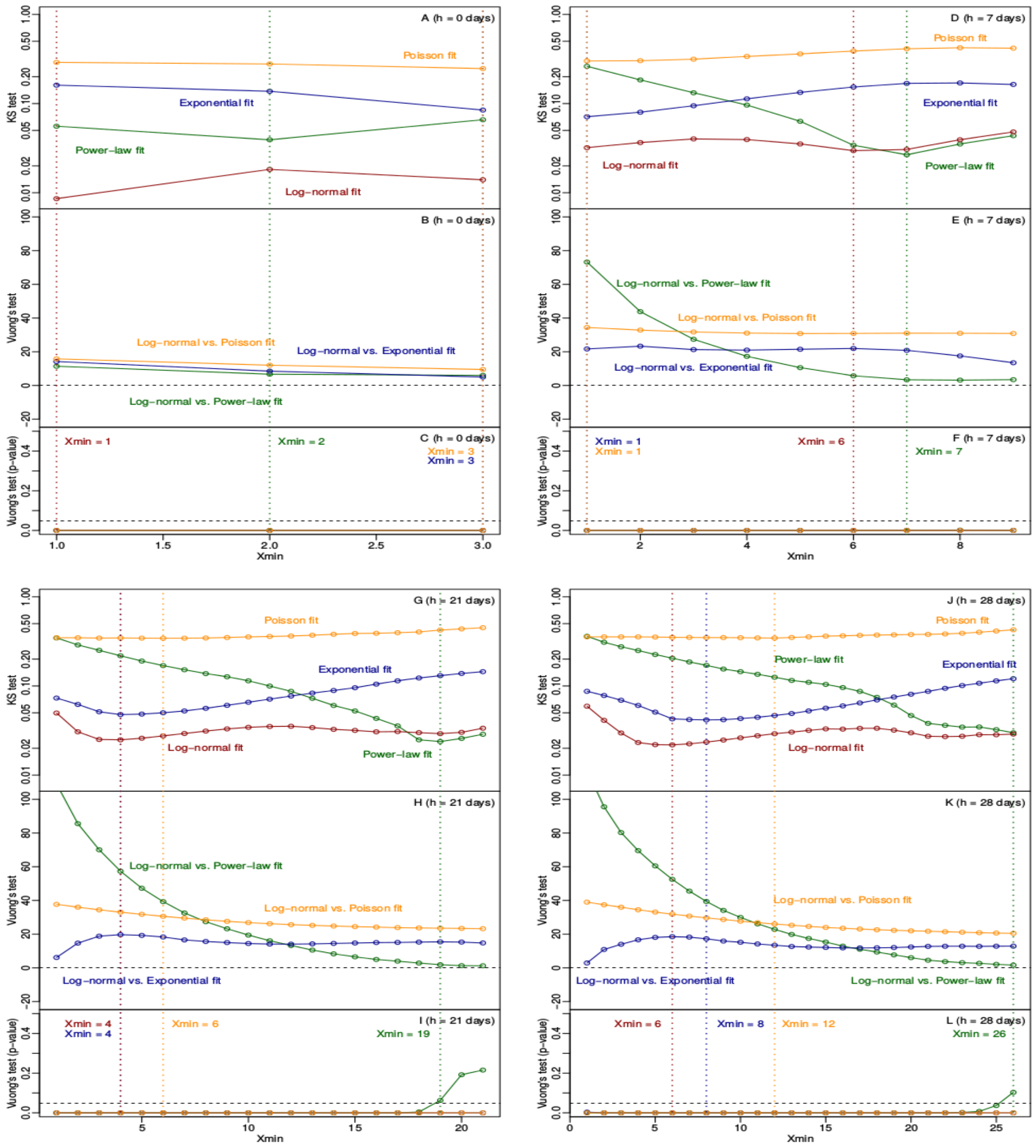

Figure S2.3: Sensitivity analysis of x_{min} for varying h values.

Comparison of four distribution functions fitting (Poisson log-normal, discrete exponential, power law, and Poisson) on the contacts per visit data, generated assuming four alternative contamination period lengths: 0, 7, 21, and 28 days. Figures show the effect of the x_{min} choice on the performance of the four distributions. Panels A-B-C: h set to 0 days; panels D-E-F: h set to 7 days; panels G-H-I: h set to 21 days; panels J-K-L: h set to 28 days. Panels A-D-G-J: Kolmogorov-Smirnov goodness-of-fit test results; panels B-E-H-K: Vuong's test results; panels C-F-I-L: Vuong's test p-value.

Number of contacts per visit – Distribution and fitting						
Contamination period (h) – days		0	7	14	21	28
# visits				20577		
#zeros		13750	2081	981	643	498
	5th quantile	1	1	1	2	2
	Median	1	4	8	11	15
#non-zeros	80th quantile *(x_{max})*	*3*	*9*	*15*	*21*	*26*
	95th quantile	8	21	29	38	46
	Max	41	71	110	144	172
**Discrete log-normal fit**						
x_{min}		1	6	2	4	6
#data between 0 and x_{min}		0	11114	1201	2492	3219
#data in the tail		20569	9442	19347	18047	17312
Parameter 1 (mean-log)		-0.63	-2.32	2.08	2.48	2.76
Parameter 2 (sd-log)		1.37	1.78	0.83	0.76	0.71
**Power-law fit**						
x_{min}		2	7	13	19	26
#data between 0 and x_{min}		3881	12620	14179	14911	15742
#data in the tail		16688	7936	6369	5628	4789
Exponent		2.31	2.72	3.02	3.19	3.40

Table S2.1: Number of contacts per visit distributions and distribution fitting.

Number of contacts generated by each visit under five different assumptions on the contamination period length (h = 0, 7, 14, 21, and 28 days). For each h value we showed: the number of visits not generating contacts (zeros), the 5th, 50th, 80^{th}(x_{max}), and 95th quantiles and the maximum of the non-zeros values, and the parameters for the two distributions providing the best fit (Poisson-lognormal and power-law).

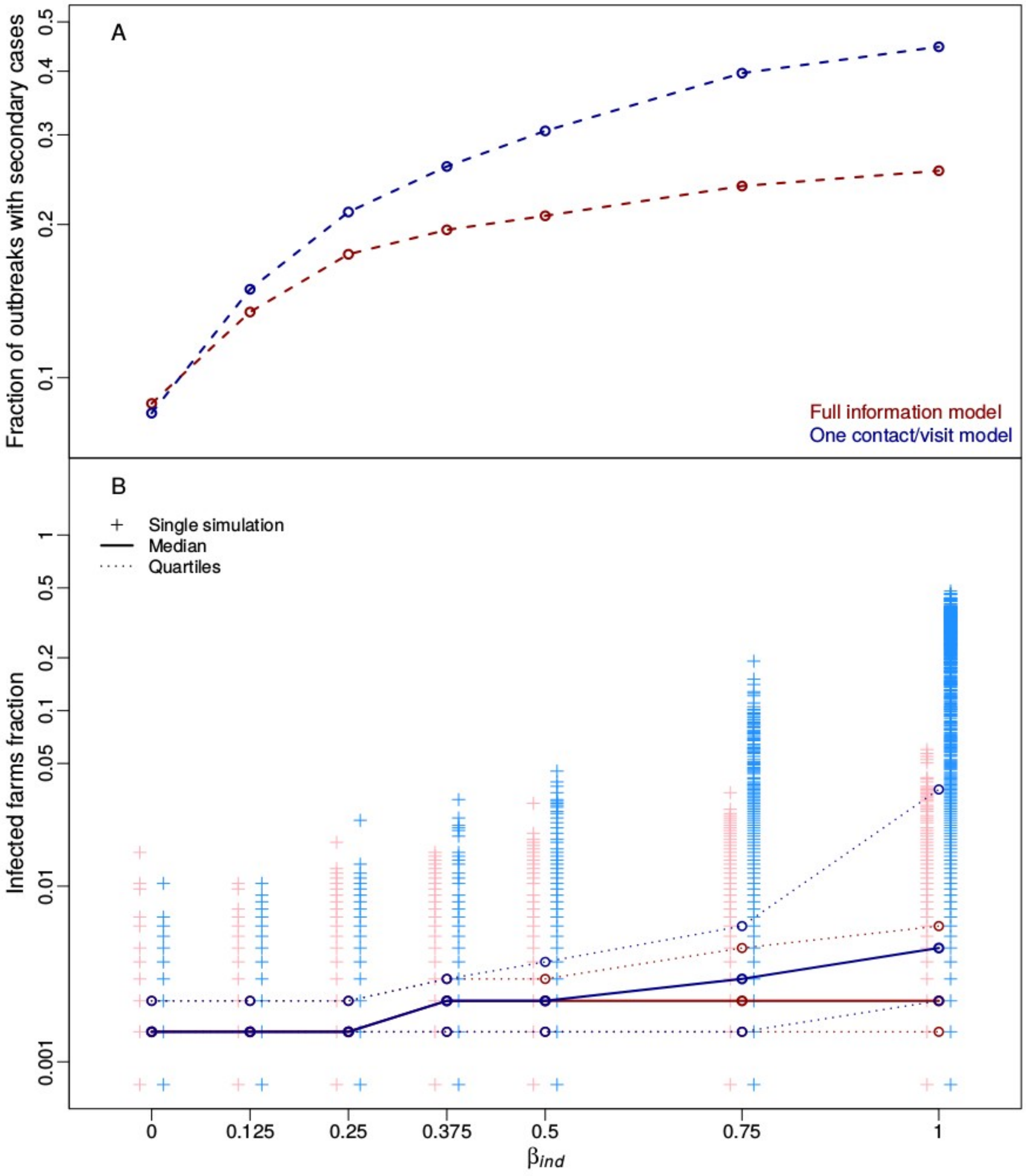

Figure S2.4: Epidemic simulations assuming one-contact-per-visit model.

Results of the simulations comparing the original model (red), the model assuming only one contact generated by each visit (blue). Conversely to Figure 2 (see main text), here h was set to 0 days. Panel A: dashed lines represents the fraction of outbreaks with secondary cases. Panel B: the distribution of the number of infected farms (crosses), with the median (thick line), and the 25th and 75th quantiles (dotted lines).

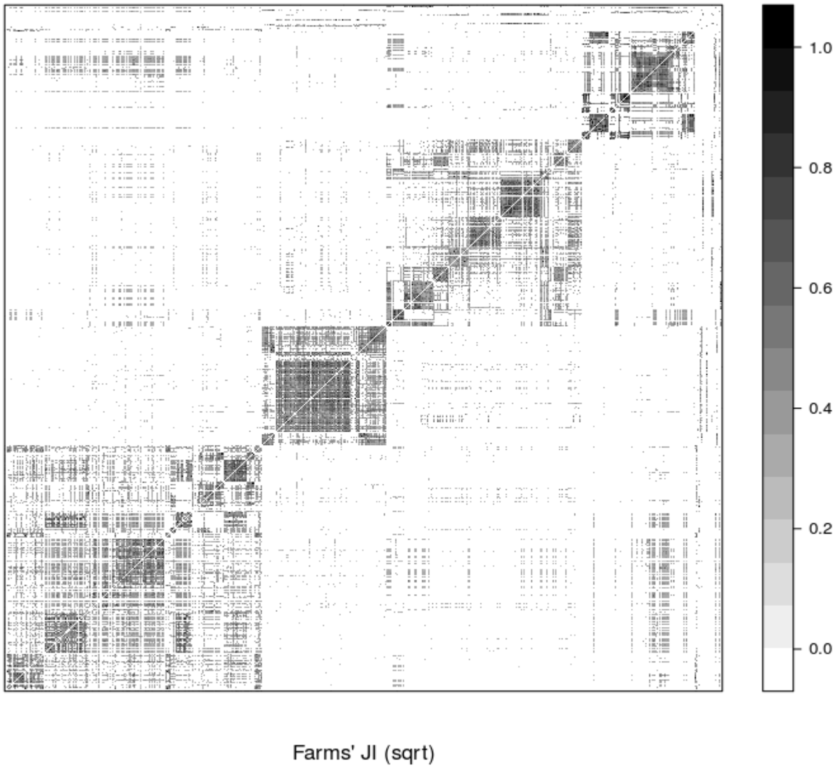

Figure S3.1: Farms-veterinarian Jaccard Index.

The matrix representing the observed values of the farms-veterinarian Jaccard Index JI_{ij} (see main text section 2.5, (3)) calculated for each pair of farms *i* and *j* (on x- and y- axes, matrix is symmetric). Grey scale represents *JI* values between 0 (white) and 1 (black), and they are square rooted for visualization purposes.

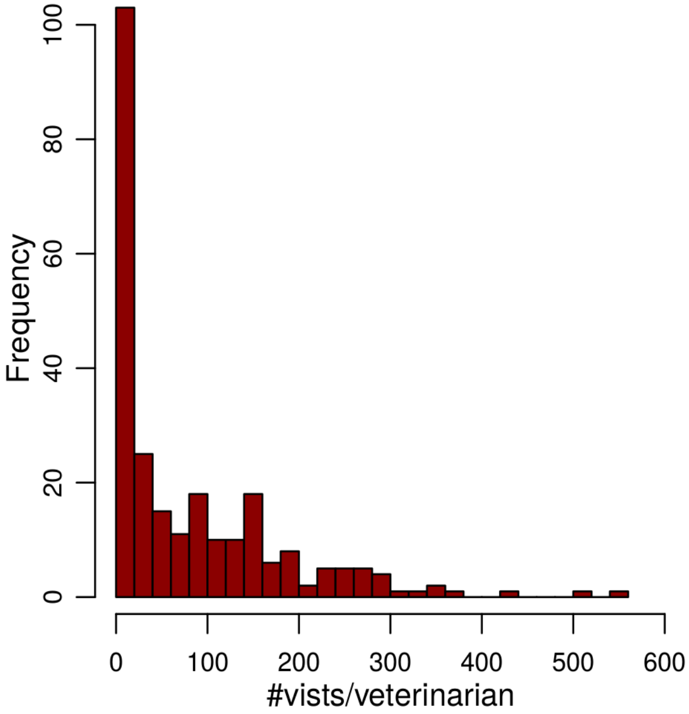

Figure S3.2: Observed visits per veterinarian distribution.

Distribution of the observed number of visits made by each of the 253 veterinarians in the Province of Parma (IT) in 2013.

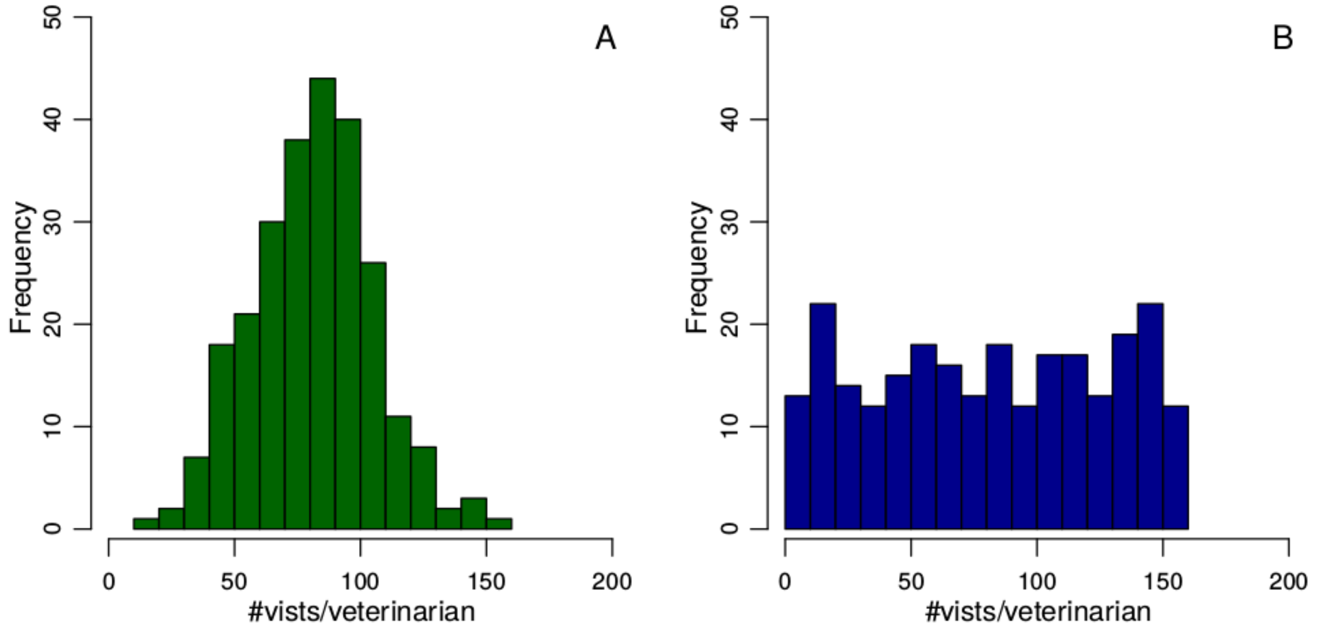

Figure S3.3: Alternative visits per veterinarian distributions.

An example of the normal (A, green) and uniform (B, blue) distributions of the number of visits per veterinarian used to test the effect of these distributions on the simulations outcome.

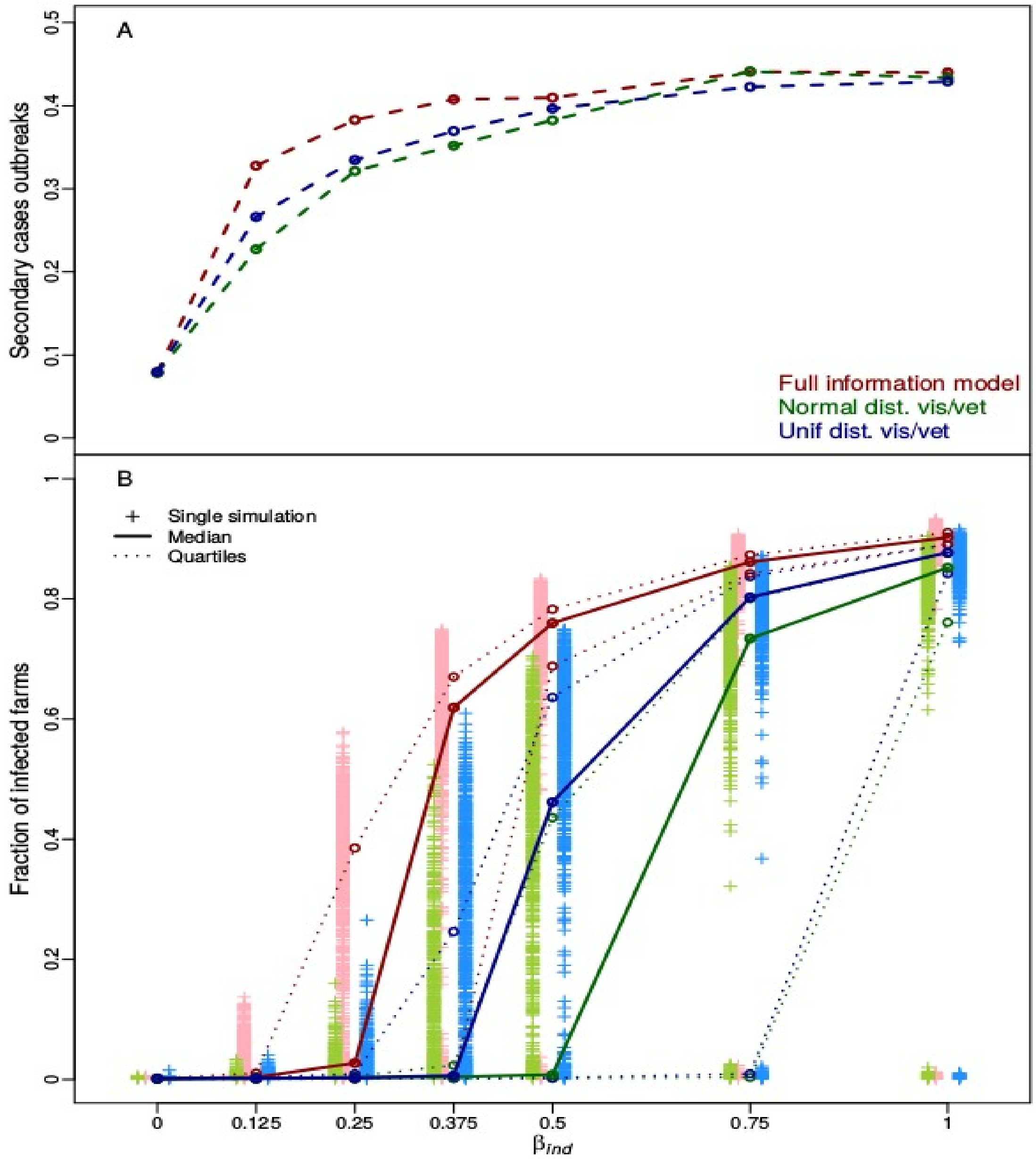

Figure S3.4: Simulations assuming alternative visits per veterinarian distributions.

Results of the simulations comparing the original model (red), and two models fed with re-built data varying the number of visits per veterinarian. We assumed normal (green) and uniform (blue) distributions, instead of the one showed in Figure S4.2. Panel A: dashed lines represent the fraction of outbreaks with secondary cases. Panel B: the distribution of the number of infected farms (crosses), with the median (thick line), and the 25th and 75th quantiles (dotted lines).
